# Protective Efficacy of Vitamins C and E on *p,p′*-DDT-Induced Cytotoxicity via the ROS-Mediated Mitochondrial Pathway and NF-κB/FasL Pathway

**DOI:** 10.1371/journal.pone.0113257

**Published:** 2014-12-02

**Authors:** Xiaoting Jin, Li Song, Xiangyuan Liu, Meilan Chen, Zhuoyu Li, Long Cheng, Hua Ren

**Affiliations:** 1 Institute of Biotechnology, Key Laboratory of Chemical Biology and Molecular Engineering of National Ministry of Education, Shanxi University, Taiyuan 030006, China; 2 College of Life Science, Zhejiang Chinese Medical University, Hangzhou 310053, China; 3 Program in Neuroscience, Harvard Medical School, Boston, MA 02115, United States of America; 4 Shanxi College of Traditional Chinese Medicine and Western Medicine Hospital, Taiyuan, 030013, China; Institute for Health & the Environment, United States of America

## Abstract

Dichlorodiphenoxytrichloroethane (DDT) is a known persistent organic pollutant and liver damage toxicant. However, there has been little emphasis on the mechanism underlying liver damage toxicity of DDT and the relevant effective inhibitors. Hence, the present study was conducted to explore the protective effects of vitamin C (VC) and vitamin E (VE) on the cytotoxicity of DDT in HL-7702 cells and elaborate the specific molecular mechanisms. The results demonstrated that *p,p′*-DDT exposure at over 10 µM depleted cell viability of HL-7702 cells and led to cell apoptotic. *p,p′*-DDT treatment elevated the level of reactive oxygen species (ROS) generation, induced mitochondrial membrane potential, and released cytochrome c into the cytosol, with subsequent elevations of Bax and p53, along with suppression of Bcl-2. In addition, the activations of caspase-3 and -8 were triggered. Furthermore, *p,p′*-DDT promoted the expressions of NF-κB and FasL. When the cells were exposed to the NF-κB inhibitor (PDTC), the up-regulated expression of FasL was attenuated. Strikingly, these alterations caused by DDT treatment were prevented or reversed by the addition of VC or VE, and the protective effects of co-treatment with VC and VE were higher than the single supplement with *p,p′*-DDT. Taken together, these findings provide novel experimental evidences supporting that VC or/and VE could reduce *p,p′*-DDT-induced cytotoxicity of HL-7702 cells via the ROS-mediated mitochondrial pathway and NF-κB/FasL pathway.

## Introduction

Dichlorodiphenyltrichloroethane (DDT), the first widely used synthetic organochlorine pesticide, was introduced all over the world to eliminate unwanted pests, and helped one billion people live free from malaria [1], [2]. However, its bioaccumulation, long-range transport and persistence in the environment properties raise the concerns about its possible long term adverse effects [3]. The public health issues caused by DDT began to emerge in the 1950s [4], [5]. Though having being banned or restricted for three decades, DDT is still being used for the control of vectors in some developing countries, which becomes one major sources of occupational exposure to pesticides [6]–[8].

DDT is now deemed as a probable human carcinogen and reportedly impaired liver cells [9]. Liver is an important detoxification organ and a special tissue, in which persistent organic pollutants metabolize and accumulate [10], [11]. Liver symptoms, associated with DDT poisoning, include hepatomegaly, liver damage and liver function disorder [12], [13]. It has been reported that oxidative stress can be used as a biomarker to evaluate damages and a possible mechanism of DDT and DDE toxicity in humans [14]–[16]. Furthermore, oxidative stress is closely associated with cell damage and apoptosis [17], [18].

As DDT may be present in livers of exposed humans and animals, it can cause liver damage via producing oxidative stress. To reduce or prevent the liver damage induced by DDT, some nature antioxidant supplements may achieve the desired effect through neutralizing the oxidative stress. Vitamin C (VC) and vitamin E (VE), as nature antioxidants, can act to overcome oxidative stress and have been shown to possess anti-carcinogenic, anti-clastogenic, and anti-mutagenic properties in a variety of *in vivo* and *in vitro* models of pesticide exposure [19]–[21]. VE, a major lipophilic antioxidant, resides mainly in the membranes, thus helps to maintain membrane stability, and it can promote the detoxification functions of liver cells [22], [23]. VC, a hydrophilic vitamin, is a very important free-radical scavenger, trapping radicals and protecting bio-membranes from per-oxidative damage [24]. In addition, VC has been reported to be detoxification to some toxic substances, such as arsenic, benzene, bacterial toxins [25], [26]. VC and VE prevent the increased free radicals induced by oxidative damage to lipids and lipoproteins in various cellular compartments and tissues [27].

Mitochondrial pathway and Fas/FasL pathway are the basic pathways in cell apoptosis. The mitochondrial apoptotic pathway is a high conservative process and can be regulated by apoptosis gene, such as Bcl-2 family and caspase family [28]. Most of the apoptosis signals are directed into mitochondria by changing the permeability of mitochondrial membrane, causing related substances to release from the mitochondria to the cytoplasm, and mediating the cell apoptosis [29]. In addition, Fas receptor (Fas) and Fas ligand (FasL) pathway is the other major and widely recognized signaling pathway triggering apoptosis [30]. Fas, as a surface receptor, causes apoptotic cell death when cross-links with FasL [31], [32]. The ligation of FasL to Fas in the cell membrane triggers activation of caspase-8, then caspase-8 transduces a signal to effector caspases, including caspase-3, −6, and −7, leading to the hydrolysis of cytosolic and nuclear substrates [33]. Moreover, the activation of nuclear factor NF-κB (NF-κB) is essential for the expression of FasL [34], [35].

The present study was undertaken to determine the possible effects of DDT on human liver cells, and additionally to investigate whether there is any preventive effect of plasma level of VC or/and VE upon DDT exposure, using human normal liver cells (HL-7702) as a test system. These results demonstrated that DDT exposure induced cytotoxicity of HL-7702 cells via mitochondria- and NF-κB/FasL-dependent pathway which were mediated by ROS. Plasma levels of VC or/and VE significantly ameliorated cytotoxicity damage induced by DDT. In addition, the present data suggest that the protective effects of VC and VE co-treatment are slightly higher than VC or VE, and the protective effect of VC in plasma levels is weaker than VE.

## Materials and Methods

### Reagents and Antibodies

*p,p′*-DDT (Sigma) was dissolved into dimethyl sulfoxide (DMSO) as stock solutions. The equal concentration of DMSO (0.3%) was added to medium for the control cells. VC and VE were supplied by Sangon Biotech (Shanghai, China). VC and VE dissolved in distilled water and DMSO, respectively. Annexin V-FITC apoptosis detection kit was purchased from Oncogene (San Diego, CA). The antibodies used in this study were as follows: antibodies for Bax, Bcl-2, p53, *p*-p53, Fas, FasL and cytochrome c were obtained from Bioworld Technology (Minneapolis, MN); antibodies for NF-κB p65, caspase-3, caspase-8 and caspase-9 were obtained from Beyotime Institute of Biotechnology (Haimen, China); α-tubulin was obtained from Sigma (St. Louis, MO, USA).

### Cell Culture

Human normal liver cells (HL-7702) were obtained from the Institute of Biochemistry and Cell Biology (SIBS, CAS, Shanghai, China) and used in assays at passage 7–25. The cell line has been widely used as a representative model of mammalian liver cells [36]–[38]. HL-7702 cells were maintained in RPMI-1640 medium (HyClone) supplemented with 10% FBS (Boster) and 1% penicillin/streptomycin (Solarbio) at 37°C in a 5% CO_2_ humidified cell culture incubator. To observe the toxicity of *p,p*′-DDT on HL-7702 cells, cells were exposed to *p,p*′-DDT at different doses (from 1 to 60 µM) over a 24 hours period. Cells with the treatments were then assayed for subsequent experiments.

### MTT Reduction Assay

After HL-7702 cells with different treatments for 24 h, the cell viability in control, DDT, VC, VE, VC+VE, DDT+VC, DDT+VE and DDT+VC+VE groups were measured by MTT assay. The final concentration of DMSO in the culture medium did not exceed 0.3% (v/v) during drug treatments. The MTT assay was performed as previously described [16]. Absorbance was measured at 570 nm using a microplate reader. Absorbance values presented by HL-7702 cells cultures in control group corresponded to 100% cell viability. Cell viability (%) was calculated using the following equation: cell viability (%)  =  (OD treatment/OD control) ×100. In addition, Cell apoptosis rate (%) was calculated using the following equation: cell apoptosis rate (%)  =  [1-(OD treatment/OD control)] ×100.

### Crystal Violet (CV) Staining Assay

The crystal violet (CV) staining assay was carried out as a confirmatory assay. 3000 cells were plated in culture medium (100 µL per well) in 96-well plates and incubated for 24 h at 37°C, 5% CO_2_. After treatment for 24 hours, viable cells were washed twice with PBS and fixed with 50 µL cold methanol (−20°C) for 10 min. After that, methanol was removed and cells were stained with 1% crystal violet for 15 min. The stained cells were washed with PBS and dissolved with 150 µL dissolved liquid (containing 96% ethanol and 1% acetic acid) at room temperature for 10 min. Absorbance was measured at 570 nm using a microplate reader. Absorbance values for untreated cells (control) corresponded to 100% viability.

### Flow Cytometric Analysis of Apoptosis

Apoptosis was tested by using the Annexin V-FITC and propidium iodide (PI) staining method. In brief, 5×10^5^ cells was collected and incubated in the buffer containing 200 µL Annexin V solutions (10 µL AnnexinV+200 µL binding buffer) and 300 µL PI (5 µL PI +300 µL binding buffer) in the dark at room temperature for 15 minutes. Untreated cells were used as the control. Then the samples were analyzed using a FAC Sort flow cytometer within 45 min after staining.

### Morphological Observation (Crystal Violet Staining)

Cell morphology was evaluated by light microscopy following crystal violet staining. Briefly, HL-7702 cells were grown on 12-well glass slides and cultured. After that time, cells were washed with PBS and fixed with cold methanol (−20°C). Then, cells were stained with crystal violet (1 mg/mL of final concentration), and incubated for 20 min at room temperature. Cells were examined with light microscope (10×). Apoptotic cells were identified by characteristic features of apoptosis (e.g. formation of membrane blebs and budding, cell shrinkage, condensed cytoplasm).

### Determination of ROS Generation

Generation of ROS was determined by two methods: fluorometric analysis and microscopic fluorescence imaging, using 2,7-dichlorofluorescin diacetate (DCFH-DA) as described by our previous method [16]. DCFH-DA was a cell-permeable probe, and was widely used to detect the ROS generation [39]–[41]. For fluorometric analysis, cells (1×10^6^ cells/well) were seeded in 60 mm dish. After treatment for 24 h, cells were washed twice with PBS and incubated in 500 µL 10 µmol/L DCFH-DA at 37°C for 30 min. Then cells were collected, and 200 µL of cells in suspension (1×10^5^ cells) were placed in a 96-well plate to assess cell fluorescence intensity, using a fluorescence microplate reader (Thermo Scientific Varioskan Flash, USA) with excitation at 488 nm and emission at 525 nm. The values were expressed as a percent of fluorescence intensity relative to control wells. A parallel set of cells (1×10^5^ cells/well), which were placed in 12-well glass slides, were analyzed for intracellular fluorescence using by a fluorescence microscope (Delta Vasion, USA) by grabbing the images at 20×magnification.

### Western Blotting

To analyse total protein expression, lysates of HL-7702 cells were prepared with the protein concentrations determined using BCA protein assay (Beyotime Biotech Inc., Nantong, China). Equal amounts of each total protein lysates were determined, mixed with 5×SDS sample buffer, boiled for 5 min and resolved by 10% SDS–PAGE then transferred onto nitrocellulose membranes. The blots were blocked for 1 h in PBS containing 5% non-fat dry milk (w/v) and incubated at 4°C overnight, then probed with antibody for 1 h at room temperature or overnight at 4°C. After washing, membranes were incubated at 37°C for 1 h with the appropriate horseradish peroxidase-conjugated secondary antibody (diluted at 1∶1000, Invitrogen). Protein loading was controlled by probing the membranes for α-tubulin protein. Immune-reactive proteins were detected using ECL western blotting detection system.

### Mitochondrial Membrane Potential (Δψm) Assay

Δψm is an important parameter of mitochondrial function used as an indicator of cell health [18]. Measurement mitochondrial membrane potential (Δψm) was performed in HL-7702 cells, according to the manufacturer's protocol for a commercial kit (KeyGEN BioTECH, Nanjing, China). For fluorometric analysis, HL-7702 cells (1×10^6^ cells/well) were seeded in 60 mm dish. After treatment for 24 h, cells were washed twice with PBS and then incubated with 1.0 µg/mL JC-1 for 15 min at 37 °C. Cells were collected, and 200 µL of cells in suspension (1×10^5^ cells) were placed in a 96-well plate to assess cell fluorescence intensity. The fluorescence was determined at an excitation wavelength of 490 nm and an emission wavelength of 590 nm. The values of relative monomer (green) fluorescence intensity were used for data presentation, which were expressed as a percent of fluorescence intensity relative to control wells. A parallel set of cells (1×10^5^ cells/well), which were placed in 12-well glass slides, were analyzed for intracellular fluorescence by a fluorescence microscope (Delta Vasion) (60×). Yellow-green represents the normal cells, while green represents the apoptotic cells.

### Immunofluorescence Assay

HL-7702 cells were grown on 12-well glass slides. After experimental procedures, the cells were washed with PBS, fixed with 4% paraformaldehyde and permeated in PBS containing 0.1% Triton. Next, cells were blocked with 3% BSA in PBS and incubated for 1 h with NF-κB p65 (1/500) primary antibody. The slides were then washed and incubated with corresponding anti-rabbit-FITC secondary antibodies (1/500). Then the nucleus was stained with DAPI for 30 min. After washing with PBS, the slides were mounted in gelvatol for confocal immune-fluorescence analysis. Images were acquired with a fluorescence microscope (Delta Vasion), at×60 magnification.

### Statistical Analysis

Statistical analysis was carried out using the SPSS 17.0 software program. Data, derived from three or four independent experiments, were presented as the mean ±standard deviation (SD). Differences among groups were tested by one-way analysis of variance (ANOVA) followed by Tukey's post hoc test. A value of *p*<0.05 was considered statistically significant.

## Results

### Effects of VC and VE on *p,p′*-DDT Induced HL-7702 Cell Viability Reduction

**Figure 1:**
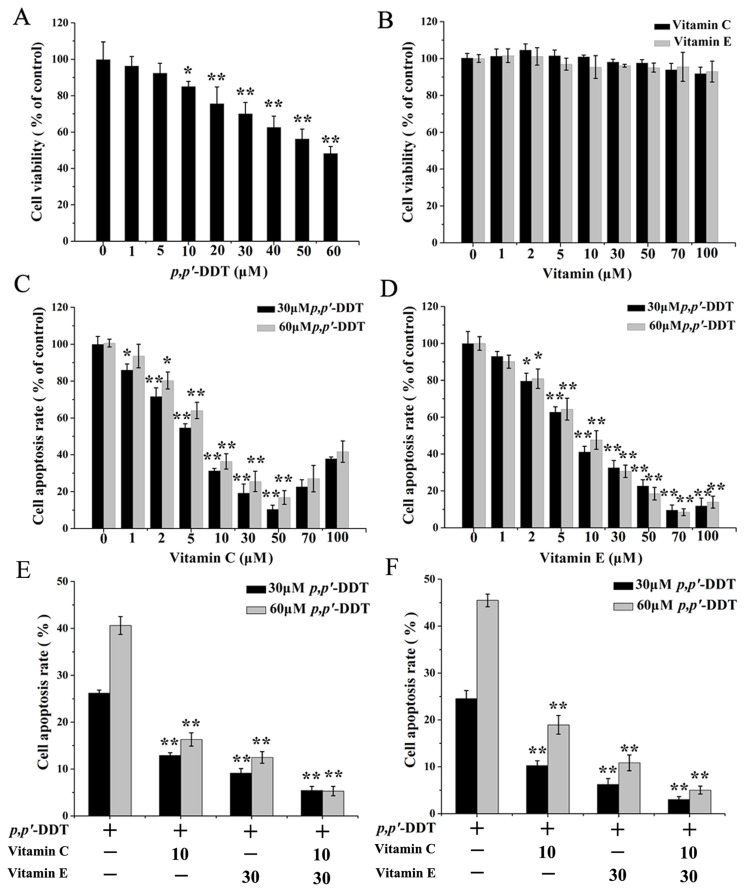
Effects of vitamin C and E on *p,p′*-DDT induced-cell viability reduction. After HL-7702 cells with different treatments for 24 h, the cell viability in *p,p′*-DDT or VC or VE group were measured by MTT assay. (A) *p,p′*-DDT group. (B) VC or VE group. Besides, the effects of VC or/and VE on *p,p′*-DDT induced-cell viability reduction were also investigated. (C) The effect of vitamin C. (D) The effect of vitamin E. (E) The effect of vitamin C (10 µM) and vitamin E (30 µM) was measured by MTT assay. (F) The effect of vitamin C (10 µM) and vitamin E (30 µM) was measured by Crystal Violet assay. The values were showed as means ± SD of triplicate determinations. An asterisk (*) represents a significant difference from controls (*p<0.05, **p<0.01).

The cell viability of HL-7702 cells was first assessed by the MTT assay (mitochondrial activity). Upon 24 h of exposure, the average percentages of cell viability were 100±9.6%, 96±5.2%, 92±5.3%, 85±2.7%, 75±9.1%, 70±6.2%, 62±6.1%, 56±5.3%, and 48±3.7% for 0, 1, 5, 10, 20, 30, 40, 50, and 60 µM, respectively, which were significant at *p,p′*-DDT concentrations ≥10 µM (*P* <0.05) (Fig. 1A). The IC50 values obtained by non-linear regression were 56 µM for MTT assays. In order to investigate whether VC or VE could play a protective role in cell viability reduction induced by *p,p′*-DDT, HL-7702 cells were exposed to VC or VE with or without the presence of *p,p′*-DDT. There were no discernible differences between VC- or VE-treated cells and control cells (Fig. 1B). Results showed that the reduction of cell viability induced by *p,p′*-DDT was markedly abolished by ≥5 µM VC or VE (*P* <0.01) (Fig. 1C and D). In addition, we take a further step to make a comparison between the protective effects of VC or VE treatment and both of them co-treatment on *p,p′*-DDT. The doses of VC (10 µM) and VE (30 µM) were chosen based on the levels of each vitamin in human plasma [42]. As shown in Fig. 1E, co-treatment with VC and VE significantly elevated the cell viability compared to *p,p′*-DDT alone group. However, the protective effect is slightly higher than VC or VE. In addition, the protective effect of VC in plasma level is weaker than VE. The crystal violet staining (cell biomass) was used as a confirmatory assay. The dose–response profiles from both assays were roughly similar (Fig. 1F). The results suggested that *p,p′*-DDT used in the present study might pose a threat to human normal liver cells, and VC or/and VE could relief the toxicity.

### Effects of VC and VE on *p,p′*-DDT Caused HL-7702 Cell Apoptosis

**Figure 2:**
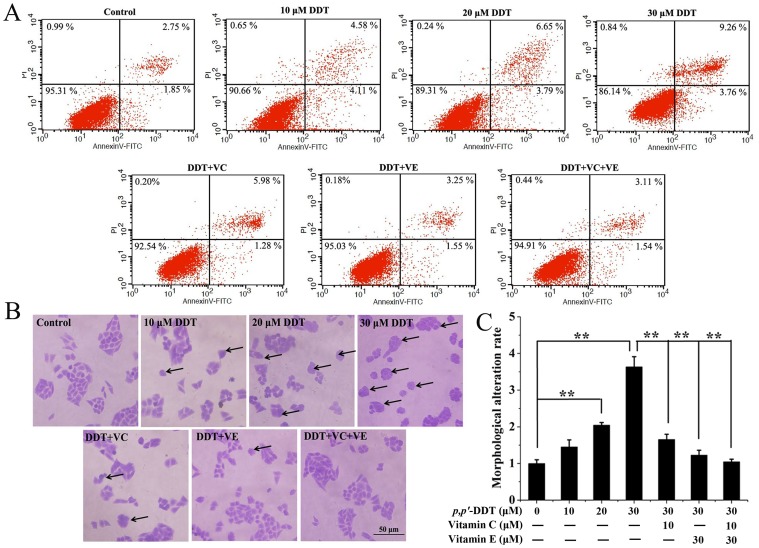
Effects of vitamin C and E on *p,p′*-DDT induced-cell apoptosis. Vitamin C (10 µM) and vitamin E (30 µM) were used to alleviate cell apoptosis induced by *p,p′*-DDT (30 µM). (A) Cell apoptosis was detected by AnnexinV-FITC/PI double staining using flow cytometric analysis. (B) Morphological changes were determined by Crystal Violet staining analysis under light microscopy (10×), and (C) the statistical analysis of representative morphological alteration was counted and the values were expressed as a percent relative to control wells. Data represent mean ± SD of three experiments, (*p<0.05, **p<0.01).

To investigate whether VC or/and VE could play prohibitive roles in apoptosis caused by *p,p′*-DDT. HL-7702 cells were incubated in various concentrations of *p,p′*-DDT (10, 20, 30 µM) and *p,p′*-DDT co-treated with 10 µM VC or/and 30 µM VE for 24 h. Apoptotic cell levels were measured through flow cytometric analysis and CV staining analysis. Flow cytometric analysis showed that *p,p′*-DDT contributed to the apoptotic cell death in a dose-dependent manner, while VC or/and VE suppressed the apoptotic rate of *p,p′*-DDT (Fig. 2A). Especially, 30 µM *p,p′*-DDT exposure induced 13.02% apoptosis compared to control cells with 4.6% apoptosis. As arrow pointed out in Fig. 2B, HL-7702 cells with *p,p′*-DDT exposure exhibited morphologically specific apoptotic characters, such as cell condensation, cell shrinkage and fragmentation. There were 3.6-folds higher in morphological changes in 30 µM *p,p′*-DDT exposure group than control group, however, VC or/and VE notably reversed the morphological alteration (Fig. 2C). Our results showed that VC or/and VE had suppressive roles in cell apoptosis induced by *p,p′*-DDT.

### Effects of VC and VE on *p,p′*-DDT Induced ROS Production

**Figure 3:**
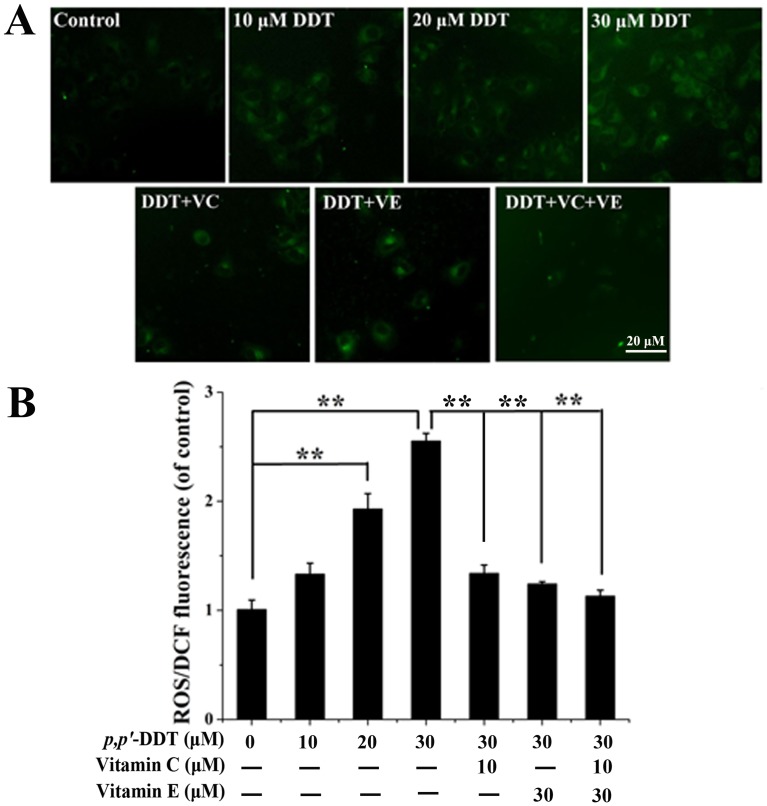
Effects of vitamin C and E on *p,p′*-DDT induced ROS production. (A) Representative microphotographs showing ROS generation induced by *p,p′*-DDT (30 µM) and the protective effects of VC (10 µM) and VE (30 µM) in HL-7702 cells. Images were captured by a fluorescence microscope (Delta Vasion). (B) Percentage alterations in the ROS generation after 24 h exposure with different concentrations of *p,p′*-DDT. Data represented are mean ± SD of three identical experiments made in five replicates. *Statistically significant differences as compared to control (*p<0.05, **p<0.01).

Oxidative stress has been implicated as an explanation behind *p,p′*-DDT toxicity, and VC or VE is a common nature antioxidant. We assumed that VC or/and VE play the prohibitive roles on *p,p′*-DDT via reducing oxidative stress, thus the generation of ROS was evaluated in HL-7702 cells. Fluorescence microscopy revealed that 10, 20, 30 µM *p,p′*-DDT induced a significant increase of intracellular ROS in a dose dependent manner, and VC or/and VE significantly repressed the ROS content (Fig. 3A). Quantitative data, using a fluorescence spectrophotometer, showed that relative intracellular ROS levels in *p,p′*-DDT group was remarkably 2.55-fold higher than that in the control group and vitamin groups alone (*P*<0.01). The content of ROS was significantly decreased in DDT + VC and DDT + VE groups compared to the DDT group (30 µM). The ROS content in DDT + VC + VE group observably declined compared to the DDT, DDT + VC and DDT + VE groups (Fig. 3B). These data illustrated that VC or/and VE could alleviate the ROS generation induced by *p,p′*-DDT.

### Effects of VC and VE on the *p,p′*-DDT Activated Mitochondrial Pathway

**Figure 4:**
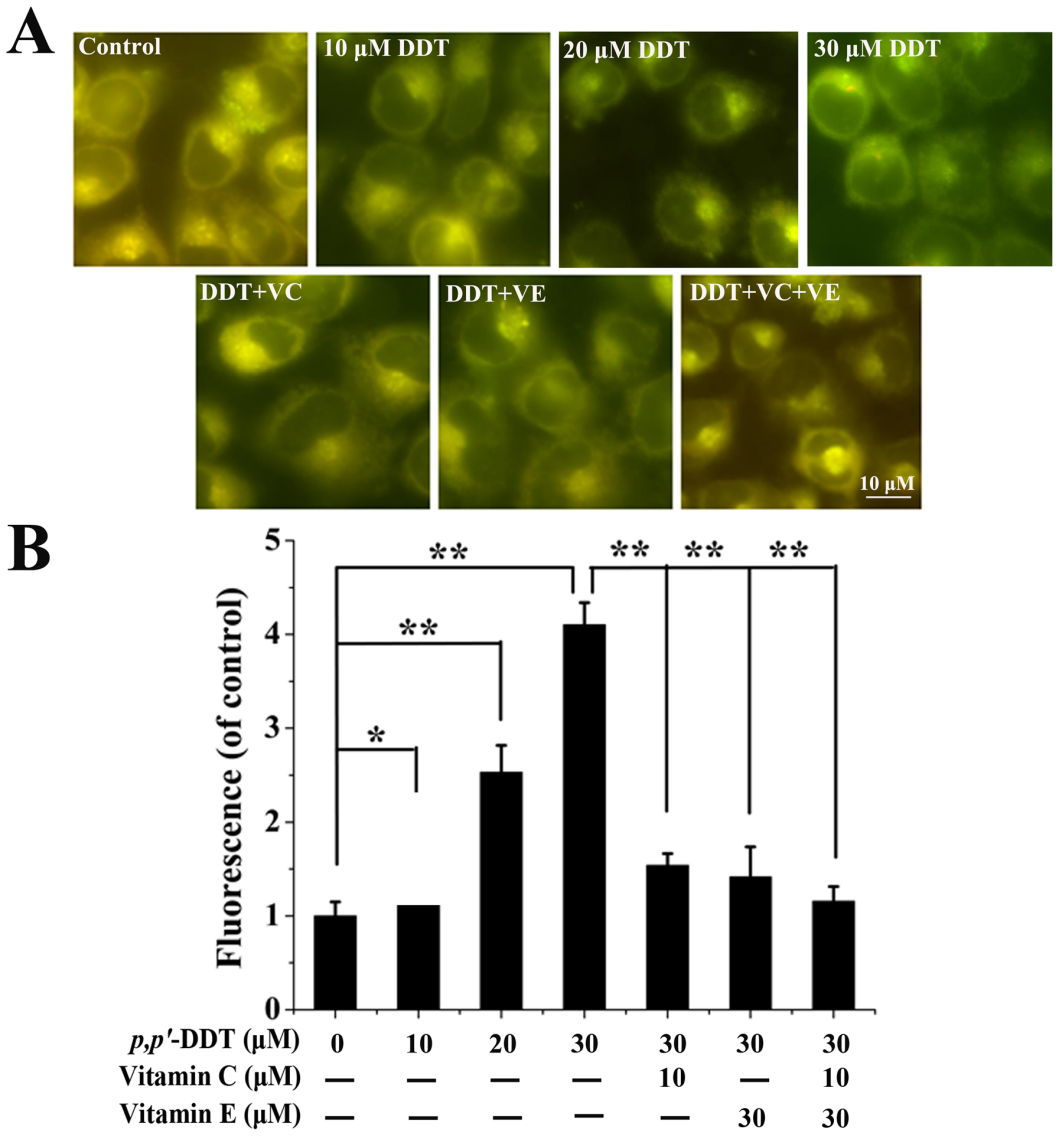
Effects of VC and VE on *p,p′*-DDT induced mitochondrial membrane potential. Cells were treated as indicated for measurement of mitochondrial membrane potential as described in Materials and Methods section. Both (A) fluorescence microscopy and (B) quantitative data revealed that VC (10 µM) and VE (30 µM) alleviated the intracellular mitochondrial membrane potential induced by *p,p′*-DDT (30 µM). Data represent mean ± SD of three experiments (*p<0.05, **p<0.01).

**Figure 5:**
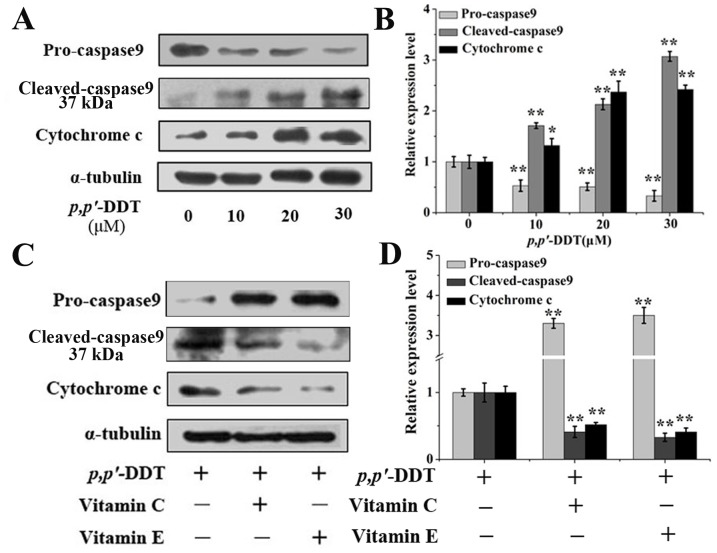
Effects of vitamin C and E on the *p,p′*-DDT induced mitochondrial pathway. Western blotting was applied to assess the activation of caspase-9 and the expression of cytochrome c, which were key roles in mitochondrial pathway. (A) Protein expressions induced by *p,p′*-DDT. (B) The grayscale scans of left western blot lines. (C) The depressant effects of VC (10 µM) and VE (30 µM) on *p,p′*-DDT (30 µM) altered proteins. (D) The grayscale scans of left western blot lines. The above blots and data were representative of at least three independent experiments with similar results. An asterisk (*) represents a significant difference from controls (*p<0.05, **p<0.01).

Mitochondrial pathway is a major pathway for apoptotic. Therefore, a further step was taken to investigate the mitochondrial pathway so as to evaluate the specific depressant mechanism of VC or/and VE. Based on Figs. 4A and B, mitochondrial potential was dramatically elevated with 10, 20, 30 µM *p,p′*-DDT treatment (*P* <0.05), suggesting the damage to the mitochondria. Interestingly, co-treatment with VC or/and VE remarkably suppressed this damage in contrast with control (*P*<0.05). Western blotting was performed to assess the activation of caspase-9 and the expression of cytochrome c, which were key factors in the mitochondrial pathway. As indicated in Fig. 5A and B, *p,p′*-DDT treatment up-regulated the expression of cytochrome c. A significant 0.33-fold decrease of procaspase-9 and 3.07-fold increase of active-caspase9 was observed after 30 µM *p,p′*-DDT treatment, indicating the activation of caspase9. However, co-treatment with VC or VE could rescue these alterations effectively (Fig. 5C and D). All these results demonstrated that *p,p′*-DDT possibly contributed to the induction of permeability transition in mitochondria which played a key role in apoptosis induction, and this phenomenon was relieved by VC or/and VE supplement.

### Effects of VC and VE on *p,p′*-DDT Altered-apoptotic Protein

**Figure 6:**
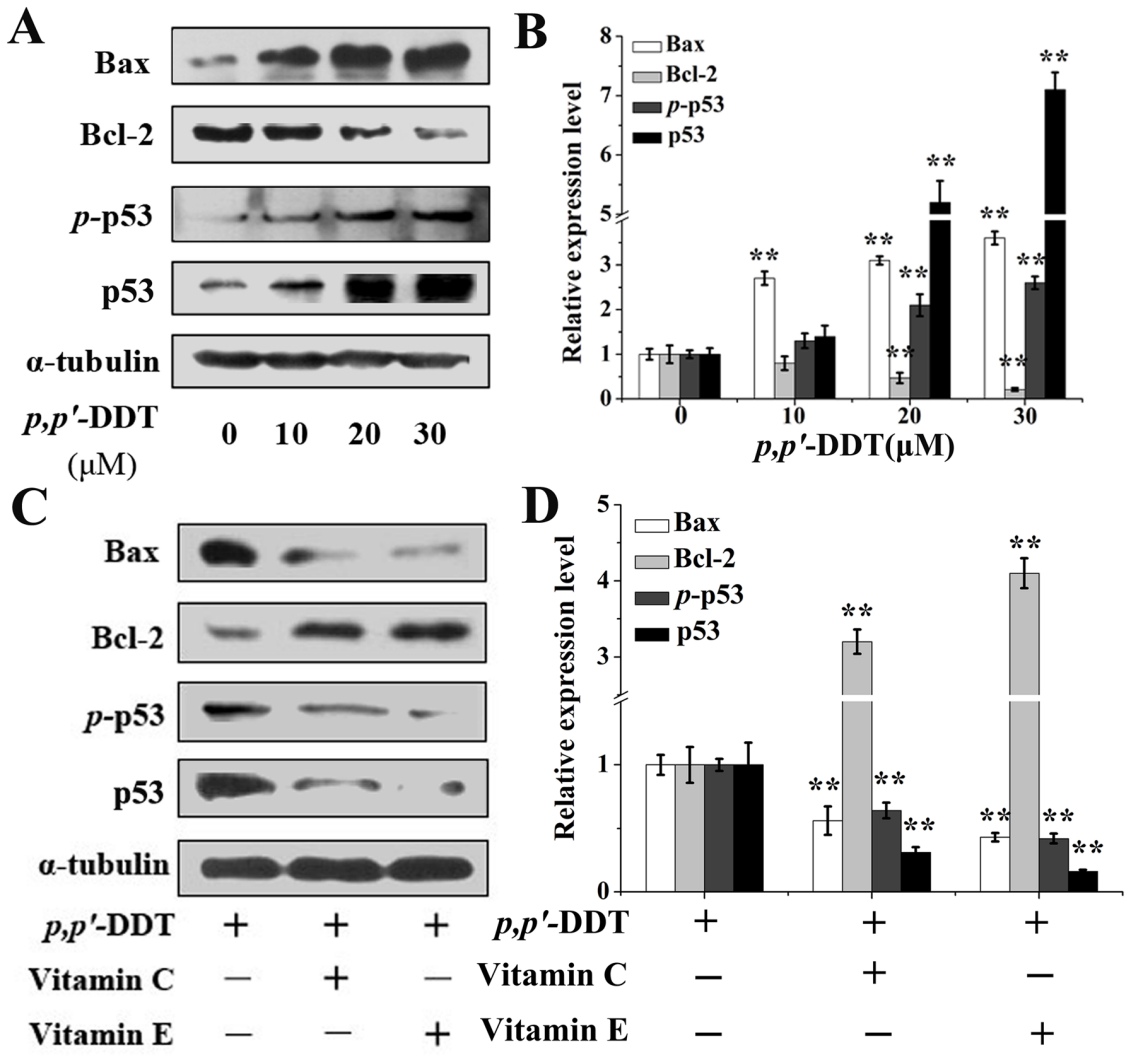
Effects of vitamin C and E on *p,p′*-DDT altered-apoptotic protein. Western blotting was utilized to analyze protein level of apoptotic genes (*Bax*, *Bcl-2* and *p53*) in HL-7702 cells. (A) Protein expressions induced by *p,p′*-DDT. (B) The grayscale scans of left western blot lines. (C) The suppressive effects of VC (10 µM) and VE (30 µM) on *p,p′*-DDT (30 µM) altered proteins. (D) The grayscale scans of left western blot lines. The above blots and data were representative of at least three independent experiments with similar results. An asterisk (*) represents a significant difference from controls (*p<0.05, **p<0.01).

Since the mitochondrial apoptotic pathway can be regulated by apoptosis genes, western blotting was utilized to analyze protein levels of apoptotic genes (*Bax*, *Bcl-2* and *p53*) in HL-7702 cells [29]. After exposed to *p,p′*-DDT at the concentrations of 10, 20 and 30 µM for 24 h, the expression levels of Bax and p53 were up-regulated, while the expression level of anti-apoptotic gene bcl-2 was down-regulated in *p,p′*-DDT treated cells as compared to control (Fig. 6A and B). Furthermore, the expression levels of *p*-p53, the activated forms of p53, were also increased. Results demonstrated that *p,p′*-DDT altered the protein levels of these genes dose-dependently. However, VC or VE had a reversed effect on these alterations. Specifically, Bcl-2 protein level, which was declined notably in *p,p′*-DDT group, could be rescued by VC or VE about 3.3 folds or 4.2 folds, respectively. In the meanwhile, Bax and p53 elevated by *p,p′*-DDT were down-regulated by VC or VE supplement (Fig. 6C and D).

### Effects of VC and VE on the *p,p′*-DDT Activated Fas/FasL Pathway

**Figure 7:**
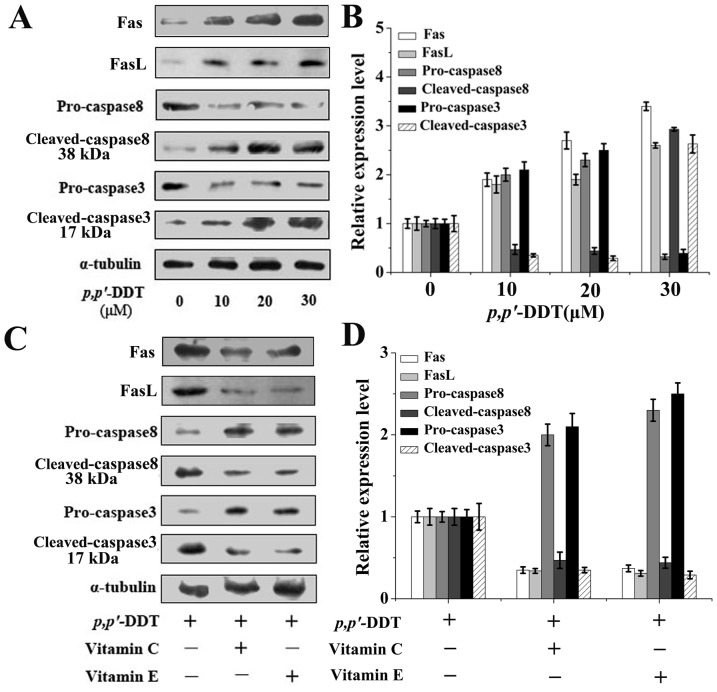
Effects of vitamin C and E on the *p,p′*-DDT activated Fas/FasL pathway. Proteins from whole cell lysates were used in western blotting to determine the expressions of FasL and Fas, along with the activation of caspase 8 and 3 in HL-7702 cells. (A) Protein expressions induced by *p,p′*-DDT. (B) The grayscale scans of left western blot lines. (C) The restraining effects of VC (10 µM) and VE (30 µM) on *p,p′*-DDT (30 µM) altered proteins. (D) The grayscale scans of left western blot lines. Data wre indicated as mean ± SD (*p<0.05, **p<0.01).

Fas/FasL pathway is a crucial signaling network triggering apoptosis. To address the effect of *p,p′*-DDT on Fas/FasL pathway, the levels of FasL and Fas were determined. *p,p′*-DDT treatment elicited an notable increase on FasL and Fas. Particularly, FasL and Fas expressions were elevated about 3.5 folds or 2.7 folds, respectively, with 30 µM *p,p′*-DDT exposure. However, the expressions of FasL and Fas were successfully attenuated by co-treatment with VC or VE (Fig. 7A and B). As caspase family members play an important role in cell apoptosis, it is also of interest to test the stimulation of caspase-8 and −3 in HL-7702 cells. As seen in Fig. 7A and B, *p,p′*-DDT treatment was shown to result in the increases of active-caspase8 and active-caspase3 protein, along with significant reductions were observed in procaspase-8 and procaspase-3, suggesting the caspase activation, respectively. Similarly, VC or VE supplement significantly counteracted these effects.

### Effects of VC and VE on *p,p′*-DDT Induced-NF-κB Activation and Translocation

**Figure 8:**
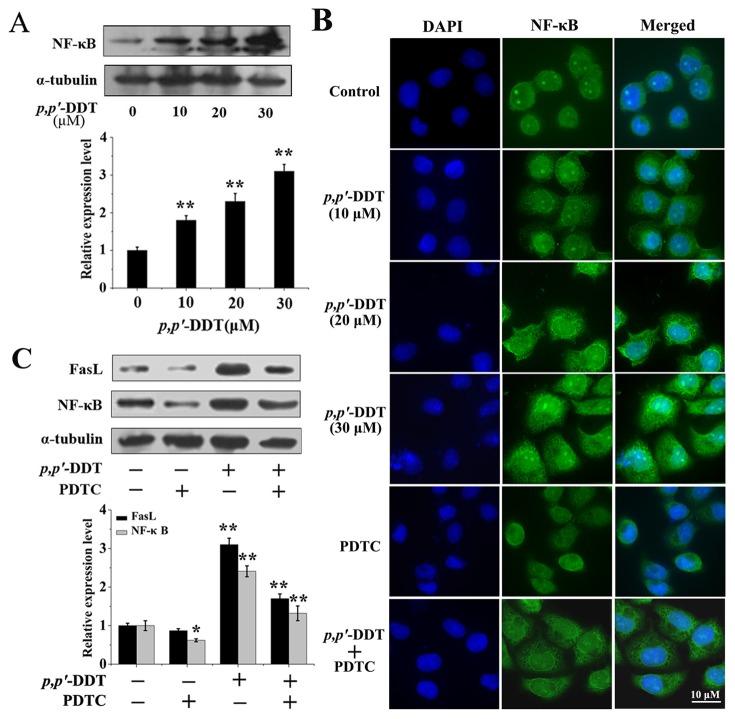
*p,p′*-DDT induced the expression of FasL via activating NF-κB p65. (A) Western blotting was carried out to exam the NF-κB expression with *p,p′*-DDT (30 µM) exposure. (B) The localization of NF-κB through indirect immunofluorescence using FITC conjugated secondary antibody. The nucleus was stained with DAPI. Cells viewed at 60×magnification. (C) The NF-κB p65 inhibitor (PDTC, 15 µM) was added to measure the effect of NF-κB p65 on FasL using western blotting.

**Figure 9:**
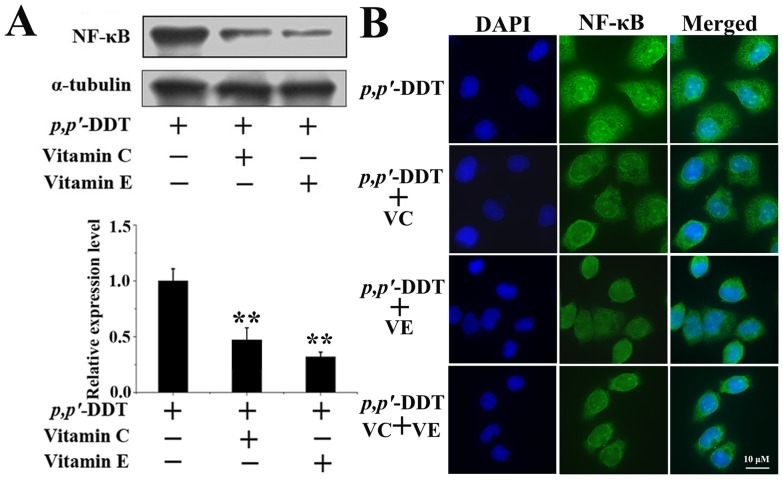
Effects of vitamin C and E on *p,p′*-DDT induced-NF-κB activation and translocation. (A) Western blotting was used to test the effects of VC (10 µM) and VE (30 µM) on NF-κB expression induced by *p,p′*-DDT (30 µM). (B) The localization of NF-κB assessed by immunofluorescence assay. The nucleus was stained with DAPI. Cells viewed at 60×magnification. An asterisk (*) indicates that the data were statistically significantly different from controls (*p<0.05, **p<0.01).

Due to NF-κB is an important factor in the regulation of FasL expression [34], [43], we hypothesized that the FasL induced by *p,p′*-DDT was mediated by NF-κB. To delineate the role of NF-κB in HL-7702 cells apoptosis, the levels of NF-κB p65 were evaluated by western blotting analysis. As indicated in Fig. 8A, *p,p′*-DDT exhibited the elevated NF-κB p65 levels in a dose-dependent manner. Immuno-fluorescence results also showed that the location of NF-κB p65 in nucleus was markedly increased in *p,p′*-DDT group. In addition, the NF-κB p65 inhibitor (PDTC) was added to measure the effect of NF-κB p65 on FasL. Strikingly, the expression of NF-κB p65 in the nuclear was inhibited by PDTC (Fig. 8B). In addition, the expression of FasL induced by *p,p′*-DDT was decreased by PDTC, suggesting that *p,p′*-DDT promoted FasL via NF-κB p65 activation (Fig. 8C). Then we assessed the effects of VC or/and VE on *p,p′*-DDT induced-NF-κB activation and translocation. As shown in Fig. 9A, *p,p′*-DDT-mediated NF-κB expression were negligible in DDT + VC, DDT + VE and DDT + VC + VE groups, due to the inhibitory effects of VC or VE on *p,p′*-DDT. Immuno-fluorescence results also showed that the location of NF-κB p65 in nucleus was prominently repressed by VC or/and VE supplement (Fig. 9B). These data indicated that VC or/and VE had repressive roles in NF-κB activation and translocation induced by *p,p′*-DDT.

## Discussion

**Figure 10:**
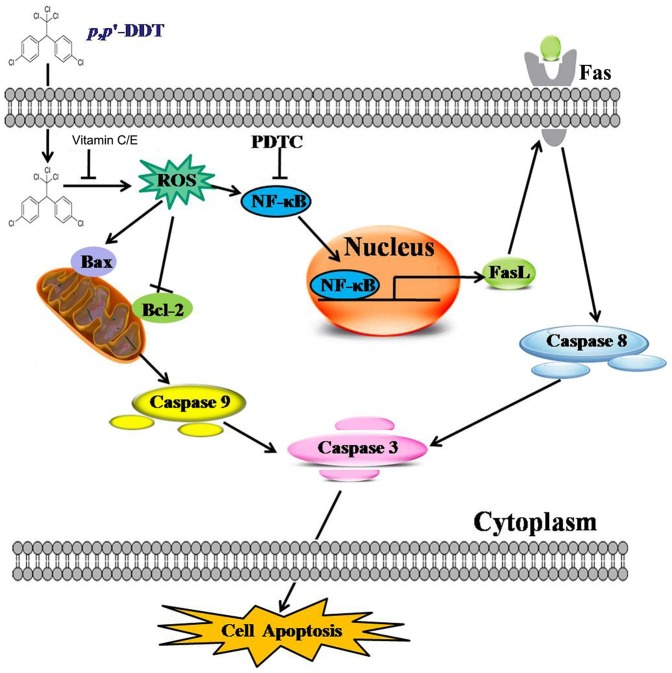
Proposed model of *p,p′*-DDT-induced signaling pathways leading to apoptosis. ROS generation might play a critical role in the initiation of *p,p′*-DDT-induced apoptosis of human liver cells through two mechanisms, one was the mitochondria-mediated pathway including elevation of ROS, decrease in ΔΨm along with the cytochrome c release from mitochondria into the cytosol, and activation of the caspase 9 and 3 in our previous study; and the other was the elevation of ROS, which resulted in the activation of NF-κB and expression of FasL, then triggered FasL-dependent pathway in the present study.

Dichlorodiphenyltrichloroethane (DDT) is a persistent organochlorine pesticide and a rodent hepatic tumor promoter for humans [1], [4]. Although there have been some literatures indicating DDT induced toxicity in liver and we have previously reported that DDT promoted the progression of liver cancer, few studies focused on the related specific mechanism involved in DDT's liver damage toxicity and the relative effective inhibitors. Therefore, in this study, we attempted to determine the effect of DDT on human normal liver cells and investigate whether there are preventive effects of VC and VE in plasma levels or not. Our study demonstrates, for the first time, that DDT exposure contributes to the elevated ROS content in HL-7702 cells, and ROS in turn serves as an activator helping to maintain NF-κB activation. Activated NF-κB complex binds to *FasL* promoter and causes robust increases in FasL levels in HL-7702 cells. Then FasL acts on Fas receptor to trigger caspase activation. At the same time, ROS induces the mitochondrial potential and contributes to the apoptosis. However, VC or/and VE supplement significantly counteract the ROS, thus eliminate the liver toxicology induced by DDT. These findings suggest VC or/and VE can reduce *p,p′*-DDT-induced cytotoxicity of HL-7702 cells via the ROS-mediated NF-κB/FasL pathway and mitochondrial pathway (Fig. 10).

DDT and its metabolites continue to be frequently found in human serum with high concentrations in malaria control areas. The mean serum concentration of *p,p*′-DDT reaches up to 90.23±102.4 µg/g and 67.8 ±31.6 µg/g in South Africa and Mexico, respectively [44], [45]. In addition, the similar concentrations (10-70 µM) of DDT were also used in other references to evaluate the toxicity of DDT [46]–[49]. The study has shown here that *p,p′*-DDT induced the cell apoptosis of human liver normal cells and VC or/and VE can relieve the toxicity. Consistent with our conclusions, many epidemiological and toxicological studies have showed that DDT contributed to hepatotoxicity in both acute and chronic forms [11], [50]–[52]. VC and VE are recognized protective agents that have been reported to repress the toxicity initiated by many different compounds. For example, VC and VE may ameliorate dichlorvos (DDVP)-induced oxidative stress by decreasing LPO in erythrocytes [53]. VC prevented the ROS generation as well as abolished almost fully the cytotoxic effect of Nickel oxide nanoparticles in human liver cells (HepG2) [54]. Harabawy *et al* reported the powerful protective potential of the VE alone and a combination of VC and VE as antioxidants against the genotoxicity and cytotoxicity of Cd, Cu, Pb and Zn in erythrocytes of O. niloticus [55].

DDT intoxication has been shown to produce oxidative stress due to the generation of free radicals in cells or tissues [14], [16]. In the present study, treatment with *p,p′*-DDT alone produced an increase in the level of ROS. Also the plasma level of VC and VE may ameliorate *p,p′*-DDT-induced oxidative stress by decreasing ROS in human liver cells at certain doses of *p,p′*-DDT. Our results are in agreement with many studies, demonstrating that VC and VE are powerful antioxidants by scavenging oxygen and preventing LPO in plasma exposed to various types of oxidative stress. Nicos Karasavvas *et al* have shown that VC protected HL60 and U266 cells from arsenic toxicity through inhibiting the generation of ROS [25]. The combination of VC and VE addition might alleviate the harmful effects of copper as copper as demonstrated by suppressing lipid peroxidation and hepatic enzymes [56]. Jianhong Zhou *et al* have reported that the supplement of VC and VE partially attenuated the arecoline-induced hepatotoxiciy in Mice by bringing the activities of alkaline phosphatase (ALP) and glutamate pyruvate transaminase (GPT) to normal levels [57]. VC, as a significant water-soluble antioxidant in plasma, can easily react with free radical in extracellular body fluids and help to reduce the effect of oxidative stress [24]. In addition, VC contributes to recycling of oxidized VE and supplying active VE fighting against LPO, thus the anti-oxidative efficiency of VE can be considerably increased by co-supplementation with VC [21], [27].

The study has indicated here that *p,p′*-DDT activated NF-κB/FasL pathway and mitochondrial pathway which were mediated by ROS. ROS derived from external sources poses a constant threat to cells as they can result in cell apoptosis, severe damage to DNA, protein, and lipids [58]. Mitochondria plays an important role in apoptosis and mediated by direct or indirect ROS action [59]. ROS activated Fas receptor-mediated apoptosis. Moreover, besides induction of apoptosis, ROS have also been shown to be involved in induction of both Fas receptor and Fas ligand genes [18]. Kasibhatla, S *et al* have shown that DNA damaging agents induced expression of Fas ligand and subsequent apoptosis in T lymphocytes via the activation of NF-κB [60]. In addition, there were many toxicological studies indicated that DDT or its metabolite contributed to cell apoptosis via FasL/Fas pathway and mitochondrial pathway. For example, Meirong Zhao *et al* reported that the enantioselective apoptosis caused by DDT might involve three signaling pathways via caspase 3, tumor protein 53 (p53) and NF-κB [61]. *p,p′*-DDE led to apoptosis of cultured rat Sertoli cells via mitochondria-mediated or FasL-Dependent Pathway [49], [62].

DDT is known to lead to rodent hepatic tumors. Nevertheless, the identification of potential action of DDT on normal liver may assist in the assessment of the toxicity of DDT to humans and wildlife. Taken together, our study indicates the novel points that plasma levels of VC or/and VE supplement can alleviate the cytotoxicity induced by *p,p′*-DDT, and its mechanism of action possibly involves the regulation of ROS production and NF-κB/FasL-dependent pathway. Moreover, we compare the protective effect of VC, VE and VC+VE, finding that the suppressive effect of VC+VE on *p,p′*-DDT is higher than VC or VE alone, while VE is slightly higher than VC. The results contribute to explaining the damage effects of *p,p′*-DDT contaminants on the liver and provide the effective inhibitors to alleviate the cytotoxicity induced by *p,p′*-DDT.
